# Driving Transparency and Efficiency in Value-Based Care Through Bundled Episodes and Technology

**DOI:** 10.7759/cureus.97752

**Published:** 2025-11-25

**Authors:** Amol Kodan

**Affiliations:** 1 Public Health, Monroe University, New York City, USA

**Keywords:** care coordination, episode-based care, healthcare transparency, patient trust, value-based care

## Abstract

The current healthcare system often feels confusing and fragmented for patients, with unclear costs, repeated tests, and delays in care. The traditional fee-for-service (FFS) model in healthcare, which reimburses providers for each individual service regardless of necessity or outcome, has long fostered inefficiencies, redundant testing, and administrative burden. This approach often leads to fragmented care, delayed diagnoses, and a breakdown in patient trust, especially when costs are unclear and care decisions lack transparency. In response, there has been a national shift toward value-based care (VBC) and episode-based alternative payment models (APMs), which aim to prioritize quality, coordination, and outcomes over volume. However, the success of these models hinges on one essential principle: transparency. This article explores how transparency, when applied at clinical, financial, and operational levels, can rebuild patient trust, reduce unnecessary procedures, and improve accountability across healthcare systems. The integration of advanced technologies, particularly artificial intelligence (AI), further enhances the scalability and effectiveness of transparency initiatives. This article advocates for a healthcare system where decisions are clear, billing is understandable, and care is coordinated, with transparency and technology working hand in hand to achieve lasting reform.

## Introduction

For many patients, the U.S. healthcare system is not a path toward healing but a maze of delays, paperwork, opaque denials, and unexpected costs [1,2]. While the clinical capabilities of the system are often praiseworthy, the patient experience falls short of expectations, as it is marred by overwhelming, complex paperwork, a misaligned reimbursement structure, and fragmented services. This is specifically the classical experience of the system, which relies heavily on the fee-for-service (FFS) model, which rewards volume over value [3]. The traditional FFS model bills each medical service separately, regardless of necessity. The lack of real-time data sharing, opaque authorization rules, variable quality measures across payers, and inconsistent episode definitions have all impeded true transparency. This structure incentivizes increased service volume rather than better care, resulting in repeated tests, confusing bills, and poor coordination among providers. The lack of transparency and unpredictable insurance processes erode patient trust.

However, the way ahead is paving the way for a major shift. Healthcare leaders and insurance companies are transitioning toward value-based care (VBC) and episode-based payment models, where the focus shifts from the volume of care provided to its effectiveness. These newer models aim to reward quality, coordination, and good outcomes, rather than sheer volume. Still, for this shift to truly work, it has to be built on a clear and honest foundation, which is transparency [4]. For this transformation to succeed, it must be grounded in transparency, ensuring that patients, providers, and payers are all aligned on how care is delivered, evaluated, and paid for.

Transparency in healthcare refers to making clinical decisions, costs, quality measures, and administrative rules visible, understandable, and consistent for patients, providers, and payers. It refers to the clear, accessible, and timely communication of information about clinical decisions, care processes, outcomes, pricing, and payment policies shared in a manner that patients, providers, and payers can understand and use to make informed decisions. Clinically, financially, and operationally transparent systems enhance trust, minimize confusion, and facilitate coordinated care. VBC builds on this foundation by shifting reimbursement away from the traditional FFS model toward one that rewards quality, outcomes, and efficiency. Instead of paying for every test or visit, VBC incentivizes prevention, coordination, and improved patient health, aligning financial incentives with better clinical results. Within VBC, bundled or episode-based payments offer a single, fixed reimbursement for all services related to a specific condition or procedure. By tying payment to the entire care episode rather than individual services, bundled models reduce redundancy, promote teamwork, and strengthen accountability, making transparency essential for success. Although reimbursement is delivered as a single episode-based payment, transparency requires that the underlying services remain clearly itemized so that patients and providers understand what the bundle includes. Additionally, all included services must be clearly itemized, allowing patients and providers to understand what the bundle covers and how costs are allocated.

This paper explores how transparency can transform episode-based care into a trustworthy, efficient, and equitable system for all stakeholders, and how we can adapt to a model in which patient outcomes are a valued priority. It functions as a conceptual policy analysis supported by technical evidence, aimed at outlining how transparency, augmented by AI, can operationalize the shift toward VBC and episode-driven care.

## Technical report

Methodology

This paper uses a literature synthesis and conceptual policy analysis to examine how transparency, VBC, and AI can be integrated to reform U.S. healthcare delivery. It synthesizes a targeted review of peer-reviewed studies, federal policy reports, implementation evaluation models, and recent analyses of AI-enabled administrative and clinical pathways. No new empirical data were collected; instead, findings were integrated to build a framework for how AI-enabled, episode-bundled VBC can strengthen accountability and improve patient experience.

The FFS Dilemma: Incentivizing Inefficiency and Redundancy

The core limitation of the FFS model lies in its incentive structure. Providers are reimbursed for each service rendered, including imaging scans, lab tests, consultations, and procedures, regardless of necessity or outcome. This disconnection between cost and value promotes overutilization, fragmented care, and a reactive rather than preventive approach to health management.

The Institute of Medicine (2012) estimated that nearly 30% of total U.S. healthcare spending is wasteful, driven by unnecessary procedures, administrative overhead, and redundant diagnostics. One of the clearest manifestations of this is seen in imaging step therapy [2]. Insurers commonly require patients to undergo lower-cost imaging, such as X-rays, before approving higher-value modalities like MRIs, regardless of clinical appropriateness. While designed to reduce spending, these prior authorization (PA) processes often result in delayed care, increased patient anxiety, additional radiation exposure, and duplicated services. Health Affairs (2020) reports that imaging authorizations under FFS introduce delays of 6 to 14 days per case. Furthermore, MedPAC's 2024 Report to Congress highlighted similar inefficiencies in Medicare step-therapy protocols, which often demand sequential diagnostics, even in time-sensitive scenarios [5]. The paradox is that efforts to reduce immediate costs often lead to higher downstream expenditures and lower care quality. Authorization algorithms, proprietary and opaque, further complicate the decision-making process. Clinicians face a dilemma: which to pursue, what is clinically justified, or comply with payer protocols that may contradict best practice. This opaque system degrades provider morale and undermines patient care.

Value-Based and Episode-Based Models: Realigning Incentives

VBC aims to realign incentives by tying reimbursement to outcomes rather than service volume. Providers are rewarded for improving health, avoiding complications, and managing care efficiently over time. Among VBC strategies, episode-based payment models are particularly promising. These models bundle all services related to a specific condition or procedure into a single, risk-adjusted payment. For example, a hip replacement episode would encompass diagnostics, surgery, inpatient stay, rehabilitation, and follow-up care across all providers involved.

**Figure 1:**
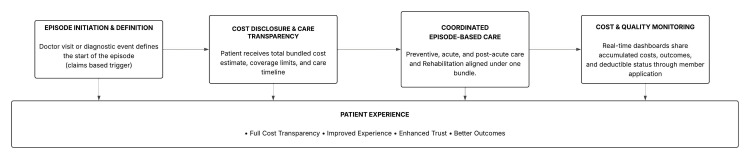
Transparency in the algorithm for value-based care

This approach promotes coordinated care, a unified payment structure, and enhanced patient outcomes. The CMS Innovation Center's 2022 Report to Congress found that episode-based models with transparent quality metrics and robust data sharing consistently outperformed FFS models in both patient satisfaction and clinical outcomes [6]. However, it is imperative to build these models on pillars of transparency and care. Figure 1 recapitulates the flow diagram that focuses on transparency in the algorithm of VBC and its outcome. This figure represents a conceptual future-state model illustrating how transparency and AI-enabled coordination could function in episode-based care, rather than the current state of system operations.

Transparency: The Foundation of Trust and Better Care

For VBC to truly succeed, transparency cannot be an afterthought, and it must be front and center. When patients, providers, and health systems have access to clear and understandable information, it fosters trust, reduces confusion, and makes care more efficient. But transparency is not just one thing; it must occur at three key levels: clinical, financial, and operational. Clinical transparency is where doctors and healthcare teams require clear, evidence-based guidelines to inform their care decisions. Financial transparency means patients deserve to know the cost of their care before they receive it, including upfront information on what their insurance covers and what they will be expected to pay out of pocket. Operational transparency refers to systems in which healthcare providers openly share key performance data, such as hospital readmission rates, complication rates, and patient satisfaction scores. When this information is accessible, it holds organizations accountable and helps patients choose providers based on real patient-centered quality results.

Transparent systems reduce administrative burdens, promote shared decision-making, and enable patients to engage more meaningfully in their care journey.

AI as a Catalyst for Transparent, Scalable Reform

Bringing real transparency to healthcare is not just a policy goal; it is a practical challenge that requires smart tools to make it work. This is where AI steps in as a powerful catalyst [7,8]. Spurred by advances in processing power, memory, storage, and an unprecedented wealth of data, computers are being asked to tackle increasingly complex learning tasks, often with astonishing success. Computers have now mastered a popular variant of poker, learned the laws of physics from experimental data, and become experts in video games with tasks that would have been deemed impossible not too long ago. In parallel, the number of companies centered on applying complex data analysis to varying industries has exploded, and it is thus unsurprising that some analytic companies are turning attention to problems in healthcare. The purpose of this review is to explore what problems in medicine might benefit from such learning approaches and use examples from the literature to introduce basic concepts in machine learning. It is important to note that sufficiently large medical datasets and effective learning algorithms have been available for many decades, yet, although there are thousands of papers applying machine learning to medical data, very few have contributed meaningfully to clinical care. This lack of impact stands in stark contrast to the enormous relevance of machine learning to many other industries. Furthermore, strict privacy regulations, HIPAA regulations, and limited data sharing across institutions restrict access to large, representative datasets and create an additional barrier to robust machine learning development and validation in the complex healthcare ecosystem. Thus, part of my effort will be to identify what obstacles there may be to changing the practice of medicine through statistical learning approaches and discuss how these might be overcome [7].

## Discussion

While transparency is a value, AI is the enabler. AI is a tool that operationalizes transparency in real time and at a larger scale [7]. Far from being a futuristic add-on, AI has the potential to make transparency a reality across every layer of care, turning data into clarity and complexity into coordination. When integrated into episode-based care models, AI can group related services into one cohesive picture, streamline prior authorizations by aligning clinical data with guidelines, and detect billing irregularities before they become costly errors [4]. Just as importantly, these systems must be explainable and ethical, which ensures that decisions are transparent, consistent, and free from bias. The goal is not to eliminate human judgment but to enhance it by leveraging AI to support fair, efficient, and trustworthy care delivery. The goal is not to automate away human oversight but to support human decision-making with consistent, transparent logic.

As this momentum gains pace to carve a revolutionary change in our healthcare towards transparency, it is critical to discuss possible real-world scenarios and challenges in this path to transparency in action.

Patients in these systems feel more supported and informed, experience less anxiety, and report greater satisfaction, ultimately leading to better health outcomes. Early evaluations show that opaque FFS authorization processes can delay clinically appropriate imaging by an average of 6 to 14 days, a barrier that value-based, AI-enabled pathways have begun to shorten through more streamlined approvals [5].

Despite the inertia of initiating and potential challenges as we transition to a transparent, AI-enabled, episode-based care model, a well-regulated implementation could revolutionize the healthcare system. Regulatory mandates for standardization (e.g., from ONC and CMS) and an effective training system can overcome hurdles such as data interoperability stemming from fragmented EHRs and payer systems that hinder real-time episode mapping [7,9]. Governance must be focused on ethics, with AI models being transparent, bias-mitigated, and continuously monitored to prevent harm. Payer resistance may be a hurdle, as some insurers may be reluctant to relinquish opaque control mechanisms. But in this health revolution, transparent contracting models and aligned incentives are necessary. It is important to ensure digital inclusion through patient-facing tools that are accessible, intuitive, and culturally appropriate to ensure equity in transparency efforts.

The transparency dividend: reducing fraud, cost, and burden

The U.S. healthcare system loses an estimated $100 billion annually to fraud and abuse [9,10]. Much of this waste thrives in opaque, fragmented environments. Transparent systems make care episodes and payment rules visible, exposing fraud and inefficiency before payments are made. For example, duplicate claims are flagged before approval, or upcoding is detected via predictive analytics. Unbundled services are automatically grouped and corrected. Patients experience fewer billing errors and faster dispute resolution. Providers face fewer arbitrary audits and clearer compliance standards. Payers see reduced losses and improved oversight. This is the transparency dividend in a healthcare system where visibility fosters integrity, efficiency, and trust.

Transparency in healthcare is not just about data visibility alone; it is about empowerment. When patients, providers, and payers operate in a shared, transparent environment where AI augments, not obscures, clinical and administrative decisions, the system becomes safer, smarter, and more focused on quality outcomes [11,12].

A recent review highlights that AI in value-based healthcare can operate through agency, automation, and augmentation pathways, which drives to better quality outcomes.

Case scenarios

The daily patient stories echo the need for transparency in action. For example, consider a hypothetical based on a real-life scenario where a patient has suspected osteosarcoma. Under FFS, the provider orders an MRI, but coverage is denied pending an X-ray. The patient undergoes the X-ray, waits for results, resubmits for an MRI, and faces further delays. Diagnosis is delayed by up to two weeks, increasing patient distress and potential harm. On the other hand, under VBC with AI, the Episode AI Engine reviews the clinical data, confirms that an MRI is indicated, and authorizes it immediately. The scan is performed within 24 hours, and treatment begins promptly. Studies have also underscored the value of AI in scaling musculoskeletal care at a larger scale [13,14]. Providing VBC using AI has proven its value across fields of healthcare, including cardiovascular prevention [15].

The path to transparency is challenging, with many hurdles to bring it into reality, but the struggle to achieve it is worth the pain when the outcomes are reduced waste, restored trust, and improved care. Such a system defines the future of equitable, transparent, and quality outcome-oriented healthcare.

Future research should focus on generating empirical data through pilot implementations, quantitative analyses, and system-level performance metrics to validate the impact of transparency and AI-enabled episode-based care. Such studies will be essential to strengthen the evidence base and translate this conceptual model into measurable, real-world outcomes.

## Conclusions

The core challenge in U.S. healthcare is not a lack of innovation or investment, but rather a lack of transparency. The FFS model prioritizes volume over value, resulting in redundancy, delays, and mistrust. As value-based and episode-based models expand, transparency must guide these reforms. Combined with ethical, explainable AI, transparency becomes an actionable framework for decision-making. An AI-enabled Episode Engine can unify care, clarify decision-making, detect fraud, and simplify administration, shifting the system from reactive oversight to proactive trust-building.

The path forward is to replace opacity with transparency and restore integrity. To improve care and reduce costs, healthcare must be transparent in every decision, episode, and dollar spent.
